# Eight weeks of high‐intensity interval training is insufficient to prevent sitting‐induced endothelial dysfunction and microvascular impairment

**DOI:** 10.1113/EP093646

**Published:** 2026-04-27

**Authors:** Nobukazu Kasai, Hayato Ihara, Motoyuki Iemitsu, Takuma Morishima

**Affiliations:** ^1^ Faculty of Health and Medical Sciences Aichi Shukutoku University Nagakute Aichi Japan; ^2^ Faculty of Sport and Health Science Ritsumeikan University Kusatsu Shiga Japan; ^3^ Faculty of Liberal Arts and Sciences Chukyo University Nagoya Aichi Japan

**Keywords:** endothelial function, HIIT, microvascular function, prolonged sitting, shear rate

## Abstract

Prolonged sitting disrupts lower‐limb endothelial and microvascular function, likely via reduced shear stress and blunted microvascular reactivity. We examined whether Tabata‐style high‐intensity interval training (HIIT) mitigates this sitting‐induced vascular dysfunction. Twenty‐two healthy young adults (age 20.3 ± 0.8 years) were randomly assigned to a control group (*n* = 11) or a training group (*n* = 11); each group comprised seven men and four women. Participants completed 8 weeks of HIIT (4 sessions/week) using a cycle ergometer and bodyweight exercises. Before and after the intervention, participants underwent a 3 h period of sitting during which popliteal artery flow‐mediated dilation (FMD) and reactive hyperaemic blood flow were assessed; plasma nitrate/nitrite and endothelin‐1 were also measured at rest. Sitting reduced popliteal artery blood flow and shear rate in both groups before and after training (*P* < 0.05). Popliteal artery FMD decreased after sitting at baseline in both groups and remained lower after sitting in the training group post‐intervention. However, post‐sitting FMD at week 8 was higher in the HIIT group than in controls after adjustment for pre‐sitting FMD. Post‐sitting blood‐flow area under the curve was decreased by sitting in both groups before and after the intervention (*P* < 0.05), and resting nitrate/nitrite and endothelin‐1 did not change with training. These results indicate that 8 weeks of HIIT does not prevent sitting‐induced impairments in endothelial function or microvascular function. Although the HIIT maintained higher post‐sitting FMD, HIIT alone was insufficient to counteract the acute endothelial and microvascular dysfunction imposed by sitting.

## Abstract (highlights)

- **What is the central question of this study?**
  Does 8 weeks of high‐intensity interval training in recreationally active young adults protect lower‐limb conduit artery endothelial function and microvascular hyperaemia from the acute vascular effects of 3 h of uninterrupted sitting?
- **What is the main finding and its importance?**
  High‐intensity interval training improved aerobic fitness but did not prevent sitting‐induced reductions in popliteal artery flow‐mediated dilation or hyperaemic blood‐flow area under the curve. At week 8, post‐sitting flow‐mediated dilation was higher in the training group than in controls, yet 3 h of sitting still blunted vascular responses in both groups, indicating that exercise training alone is insufficient to offset the acute vascular burden of prolonged sitting.

## 1 INTRODUCTION

Prolonged sitting is a significant behavioural risk factor for cardiovascular health. Sedentary time is also positively associated with atherosclerosis (Lim et al., 2022), which primarily occurs in lower‐limb arteries (Kroger et al., 1999). Studies have indicated that uninterrupted sitting induces endothelial dysfunction as evidenced by flow‐mediated dilation (FMD) in the lower‐limb arteries (Padilla & Fadel, 2017), suggesting endothelial dysfunction in response to reduced blood flow and shear rate while sitting.

One potential mechanism underlying this phenomenon is a reduction in nitric oxide (NO) bioavailability. NO is an important endothelium‐derived vasodilator, and its production is strongly influenced by shear stress. Interventions that increase NO bioavailability, such as chronic aerobic training or acute dietary nitrate supplementation, have been shown in some studies to attenuate sitting‐induced reductions in FMD. For example, we demonstrated that habitual endurance‐trained cyclists exhibited preserved FMD responses after prolonged sitting, which may be related to higher NO bioavailability (Morishima et al., 2020b). Similarly, acute dietary nitrate intake from beetroot juice has been reported to attenuate FMD reduction following prolonged sitting in healthy adults (Morishima et al., 2022). However, evidence regarding the protective role of aerobic fitness is mixed. Other studies have reported that higher aerobic fitness does not necessarily prevent sitting‐induced endothelial dysfunction (Garten et al., 2019), and similar declines in FMD have been observed across a range of fitness levels (Daniele et al., 2026; Liu et al., 2021). These findings suggest that although NO‐related adaptations may contribute to vascular resilience, prolonged uninterrupted sitting imposes a substantial haemodynamic stress that may not be fully offset by aerobic fitness alone. This suggests mechanisms beyond conduit‐artery NO bioavailability, particularly those related to microvascular blood‐flow regulation and the hyperaemic stimulus for FMD.

Other studies have implicated additional vascular mechanisms besides NO, in particular that the ability of the microvasculature to generate blood flow during hyperaemia plays an important role in determining FMD responses. This parameter reflects the ability of the microvasculature to generate sufficient blood flow during reactive hyperaemia, which is necessary for endothelium‐dependent vasodilation (Thorn et al., 2024). Therefore, microvascular function may influence FMD, not only by contributing to the stimulus for NO release, but also through direct vascular responses. Recent studies suggest that greater aerobic fitness may be associated with enhanced microvascular function. For example, Herrod et al. (2021) reported that aerobic fitness and quadriceps microvascular function, measured by a microbubble technique, were increased following a 6‐week high‐intensity interval training (HIIT) programme in older adults. Kasikcioglu et al. (2005) found higher brachial artery microvascular function in endurance‐trained compared with untrained individuals. Moreover, Garten et al. (2019) demonstrated that passive leg movement‐induced increases in lower‐limb blood flow are positively associated with aerobic fitness in young adults. Taken together, these results suggest that individuals with higher aerobic fitness contain a more responsive microvasculature, potentially buffering against endothelial dysfunction induced by prolonged sitting; however, it is unclear how aerobic capacity‐enhancing training affects microvascular function in response to prolonged sitting and to what extent these changes contribute to the preservation of conduit‐artery function as reflected by FMD.

In this study, we determined whether training attenuates sitting‐induced endothelial dysfunction and whether improvement in microvascular function (e.g., blood flow during reactive hyperaemia) contributes to this protective effect. By assessing conduit artery and microvascular responses simultaneously, we identified integrated vascular adaptations that may counteract the negative impact of sedentary behaviour. We selected HIIT for two reasons. First, it is efficient and reliably produces large gains in aerobic capacity with lower total exercise time compared with moderate‐intensity continuous training. Therefore, it may be used for adults with desk‐based, time‐constrained routines (Tabata et al., 1996; Weston et al., 2014). Second, previous studies show that HIIT improves vascular function at multiple levels. It enhances conduit‐artery endothelial function (Ramos et al., 2015) and induces microvascular adaptation, including greater muscle capillary density and improved microvascular reactivity (Cocks et al., 2013, 2016). Based on these results, we hypothesize that HIIT can attenuate the decline in FMD observed after prolonged sitting in untrained adults.

## 2 METHODS

### 2.1 Ethical approval

The study was conducted in accordance with the principles of the *Declaration of Helsinki* and approved by the Ethics Committee for Human Experiments at Chukyo University (ID: 2024‐006). All participants provided written informed consent before participation. Participants were made aware of the intent to publish these data when providing informed consent, and participants cannot be individually identified from data published in this paper.

### 2.2 Subjects

A total of 22 healthy recreationally active men (*n* = 14) and women (*n* = 8) were enrolled. Inclusion criteria were: living independently; absence of known cardiovascular disease, diabetes, hypertension or other serious chronic disease (including no previous history of these conditions); prior experience with exercise training; and willingness to participate in the study. We selected individuals with previous training experience and without major disease because such participants were considered appropriate for safely performing HIIT. Their habitual physical activity was assessed by questionnaire prior to the intervention. Exclusion criteria were current smoking and any history of smoking. The details of the study protocol were provided, and informed consent was obtained from all subjects before participation. Menstrual cycle phase in women was not controlled for in the present study. None of the women reported using hormonal contraceptives. Owing to scheduling and logistical constraints, it was not feasible to align all testing sessions with specific menstrual phases. This should be taken into account when interpreting the data obtained from women. All participants completed the study protocol and were included in the final analyses.

### 2.3 Experimental procedures

The subjects were randomly divided into a control group (*n* = 11, men = 7, women = 4) and a training group (*n* = 11, men = 7, women = 4). There were no significant differences in the characteristics of the two groups (Table 1). Participants in the control group were verbally instructed to maintain their habitual lifestyle, including physical activity and diet, and to refrain from initiating new structured exercise or dietary programs for the 8‐week experimental period. Adherence was not formally monitored. The participants in the training group performed HIIT 4 days per week for 8 weeks (32 training sessions in total; see ‘Training procedure’ below). Maximal oxygen uptake (${\dot{V}}_{O_{2}\max}$) testing and 3 h of sitting testing were conducted before and after the 8‐week experimental period.

**TABLE 1:** Physical characteristics, ${\dot{V}}_{O_{2}\max}$ and blood parameters before and after the intervention period.

		0 weeks	8 weeks	Two‐way ANOVA
Age (years)	Control	20.3 ± 0.8		
	Training	20.3 ± 0.8		
Height (cm)	Control	167.1 ± 7.1		
	Training	168.8 ± 6.9		
Body mass (kg)	Control	64.3 ± 11.8	63.8 ± 12.2	Group: *P* = 0.88 Time: *P* = 0.04 Interaction: *P* = 0.47
	Training	65.2 ± 9.4	64.2 ± 9.3	
Muscle mass (kg)	Control	29.5 ± 5.7	29.6 ± 5.7	Group: *P* = 0.84 Time: *P* = 0.24 Interaction: *P* = 0.70
	Training	29.9 ± 6.4	30.2 ± 6.0	
Percentage fat (%)	Control	18.5 ± 5.6	17.5 ± 5.6	Group: *P* = 0.95 Time: *P* < 0.001 Interaction: *P* = 0.26
	Training	19.1 ± 6.4	17.2 ± 6.1^*^	
${\dot{V}}_{O_{2}\max}$ (mL/min/kg)	Control	43.0 ± 6.1	42.8 ± 6.4	Interaction: *P *< 0.001
	Training	44.0 ± 4.6	49.3 ± 4.9^*†^	
Plasma NOx (µmol/L)	Control	42.6 ± 11.6	43.7 ± 9.0	Group: *P* = 0.34 Time: *P* = 0.40 Interaction: *P* = 0.24
	Training	49.9 ± 13.7	43.2 ± 8.3	
Plasma ET‐1 (pg/mL)	Control	1.2 ± 0.4	1.2 ± 0.4	Group: *P* = 0.33 Time: *P* = 0.40 Interaction: *P* = 0.27
	Training	0.9 ± 0.5	1.1 ± 0.4	

Data are means ± SD. ^*^ *P* < 0.05 vs. 0 weeks within group. ^†^ *P* = 0.018 vs. 8 weeks in Control group.

### 2.4 Training procedure

The subjects in the training group completed short‐duration, Tabata‐style HIIT for 8 weeks (100% compliance), which included 32 training sessions (4 days/week). The HIIT programme was divided into two training modalities: cycling HIIT and bodyweight HIIT. Cycling HIIT consisted of seven to eight sets of 20‐s cycling on a leg ergometer (PowerMaxVIII, Konami Corporation, Tokyo, Japan) at an intensity of approximately 170% of ${\dot{V}}_{O_{2}\max}$ at 90 rpm, with a 10‐s rest between each round (Tabata et al., 1996). This protocol is commonly referred to as ‘Tabata training’. The subjects were encouraged to complete seven to eight sets. The exercise was terminated when the pedalling frequency dropped below 85 rpm for approximately 5 s, despite strong verbal encouragement. When the subjects completed >8 sets, the exercise intensity was increased by 11 W. The training sessions were supervised by the researchers. This protocol corresponds to a Tabata‐style interval structure and is described as HIIT throughout the paper for consistency with the broader literature.

To enhance feasibility and ecological validity, the four‐session‐per‐week training programme was implemented as a hybrid schedule: three supervised cycle‐ergometer sessions in the laboratory and one home‐based bodyweight interval session. This design reduced participant burden associated with four weekly lab visits while preserving the intended weekly training dose and adherence. A recent review has defined this bodyweight‐based training as a form of Tabata training, and has demonstrated its effectiveness in improving aerobic capacity (Tabata, 2025). The structure of the HIIT session, exercise duration (20 s), rest duration (10 s) and number of sets (eight sets), was the same as that of cycling training. The specific exercises were as follows:

Set 1: jumping jacks (as fast as possible for 20 s) (10 s rest)
Set 2: squat jumps (as fast as possible for 20 s) (10 s rest)
Set 3: lunge jumps (as fast as possible for 20 s) (10 s rest)
Set 4: burpee jumps (as fast as possible for 20 s) (10 s rest)
Set 5: mountain climbers (as fast as possible for 20 s) (10 s rest)
Set 6: high knees (as fast as possible for 20 s) (10 s rest)
Set 7: tuck jumps (as fast as possible for 20 s) (10 s rest)
Set 8: jumping jacks (as fast as possible for 20 s)

In a preliminary study, we confirmed that oxygen uptake during training was approximately 90% of the ${\dot{V}}_{O_{2}\mathrm{peak}}$ using a wireless automatic gas analyser (portable metabolic analyser K5; COSMED, Rome, Italy). The subjects performed bodyweight‐based HIIT sessions independently at home or in a similar environment. To monitor adherence and ensure proper execution, the subjects were asked to record videos of themselves during the training sessions.

### 2.5 Measurements before and after the intervention period

#### 2.5.1 Maximal oxygen uptake

The subjects completed an incremental pedalling test on a cycle ergometer during the initial visit (PowerMaxVIII, Konami Corporation) to assess ${\dot{V}}_{O_{2}\max}$. The test began at 50 W, and the load was progressively increased by 30 W every 2 min. The exercise was terminated once the subject attained <70 rpm for >5 s (exhaustion) or an oxygen consumption (${\dot{V}}_{O_{2}}$) plateau. Respiratory gases were collected and analysed using an automatic gas analyser (AE300S; Minato Medical Science Co., Ltd, Tokyo, Japan). The data were averaged every 30 s. The highest ${\dot{V}}_{O_{2}}$ during exercise was designated the ${\dot{V}}_{O_{2}\max}$.

#### 2.5.2 Prolonged sitting (3 h)

Testing was performed at least 48 h after the last training session to avoid the acute effects of exercise. The subjects were asked to refrain from any exercise 24 h before the study visit. They arrived at the laboratory at 8:00 am after at least 10 h of overnight fasting (no food or drink except for water). The tests were performed in a temperature‐controlled room maintained at 23–24°C. Body composition was initially scanned upon arrival at the laboratory using bioelectrical impedance analysis (InBody430, InBody Japan Inc., Tokyo, Japan). The subjects were placed in a supine position, and an automated sphygmomanometer (Omron Corp., Kyoto, Japan) was used to measure blood pressure and heart rate after a 10 min rest. A polyethylene catheter was inserted in the antecubital vein, and a baseline blood sample was collected. All vascular measurements in the popliteal artery were conducted in the right leg. The popliteal artery was selected because sitting predominantly affects lower‐limb haemodynamics distal to the thigh, and popliteal artery FMD has been widely used in previous sitting studies as a sensitive index of vascular dysfunction. The popliteal artery diameter and blood velocity were measured using duplex‐Doppler ultrasound (LOGIQe, GE Healthcare Inc., Chicago, IL, USA). A 10‐MHz linear array transducer was placed over the popliteal artery distal to the popliteal fossa. The skin was marked for probe placement to ensure all measurements were made at the same location. Simultaneous diameter and velocity signals were obtained in duplex mode at a pulse frequency of 30 MHz and corrected with an insonation angle of 60°. Popliteal artery FMD was performed in accordance with current guideline (Thijssen et al., 2019). A rapid‐inflating cuff was placed on the lower leg (midpoint between knee and ankle). Baseline haemodynamics were recorded for 2 min, and the cuff was inflated to 220 mmHg for 5 min. Continuous diameter and blood velocity measurements were recorded for 3 min following cuff deflation. The recordings for all vascular variables were analysed offline using specialized edge‐detection software in the blind‐fashion (Cardiovascular Suite, Quipu srl, Pisa, Italy). The inter‐day test–retest reliability of popliteal artery FMD in our laboratory, performed by the same investigator, has previously been reported to have a coefficient of variation of 7.9% (Morishima et al., 2020a).

After establishing the baseline FMD, the subjects were asked to stay in a seated position for 3 h. To minimize any leg movement, a study representative monitored the participants throughout the sitting period. The participants read a book or used a smartphone during the sitting period. During this time, blood pressure, popliteal artery diameter and blood velocity were measured every hour. Following this sitting period, the subjects were manually lifted and placed back into a supine position to minimize any muscle activity of the leg. The FMD assessments were subsequently repeated.

#### 2.5.3 Data analysis

Blood flow was calculated from the continuous diameter and mean blood velocity recordings at each experimental time point using the following equation: 3.14 × (diameter/2)^2^ × mean blood velocity × 60. The absolute change of the popliteal artery FMD was calculated using the following equation: absolute FMD = peak diameter − base diameter. The percentage change of the popliteal artery FMD was calculated using the following equation: %FMD = (peak diameter − base diameter)/(base diameter) × 100. Shear rate, an estimate of shear stress without blood viscosity, was calculated as 4 × the mean blood velocity/diameter. Vascular conductance was calculated as blood flow/mean arterial pressure. Hyperaemic shear rate area under the curve (AUC) up to the peak diameter was calculated as the stimulus for FMD, as previously described (Boyle et al., 2013; Thijssen et al., 2009). Data analysis was performed by an investigator blinded to group allocation.

#### 2.5.4 Blood analysis

Enzyme‐linked immunosorbent assay was used to measure plasma endothelin‐1 (ET‐1) concentrations (R&D Systems, Minneapolis, MN, USA). The Total NO and Nitrate/Nitrite Parameter Assay Kit, which is based on the Griess assay (R&D Systems), was used to measure plasma nitrate/nitrite (NOx) concentrations. Optical densities at 450 nm (ET‐1) and 540 nm (NOx) were determined using a microplate spectrophotometer (MULTISCAN FC, Thermo Fisher Scientific, Tokyo, Japan). All samples for plasma ET‐1 and plasma NOx were assayed in duplicate. The intra‐assay coefficients of variation for plasma ET‐1 and plasma NOx were 10.5% and 1.8%, respectively. Data analysis was performed by an investigator blinded to group allocation.

### 2.6 Statistical analysis

The primary outcome was the between‐group difference in post‐sitting popliteal artery FMD at week 8. No formal a priori sample‐size calculation was performed. The final sample size was 22 (11 per group). Sensitivity analyses were performed in G*Power 3.1 (α = 0.05).

A three‐way mixed ANOVA was conducted on sitting‐involved variables, with intervention period (baseline vs. week 8) and sitting (pre‐sitting vs. post‐sitting) as within‐subject factors and group (control vs. training) as a between‐subject factor. *Post hoc* multiple comparisons were performed using Bonferroni's correction (sensitivity: minimal detectable within‐between interaction *f* = 0.257; partial η^2^ ≈ 0.062 *N* = 22; assumed correlation among repeated measures = 0.50; ε = 1.0).

To determine the group differences in post‐sitting popliteal artery FMD following the intervention period, while controlling for potential covariates, an analysis of covariance (ANCOVA) was performed. The control or training group was used as a fixed factor, and pre‐sitting popliteal artery FMD, post‐sitting hyperaemic blood‐flow AUC, post‐sitting hyperaemic shear rate AUC, or post‐sitting basal diameter (pre‐occlusion diameter) was included as a covariate in separate models. The adjusted means for post‐sitting popliteal artery FMD were compared between groups (sensitivity: minimal detectable between‐group effect *f* = 0.630; partial η^2^ ≈ 0.284; *n* = 22; 2 groups; 1 covariate; conservative as the DV–DV‐covariate correlation is not modelled in this module).

Body composition, ${\dot{V}}_{O_{2}\max}$, and blood parameters were determined using a two‐way (intervention period × group) ANOVA with Bonferroni's *post hoc* test (sensitivity: minimal detectable interaction, *f* = 0.314; partial η^2^ ≈ 0.090; *n* = 22; assumed correlation = 0.50; ε = 1.0). ANOVA and ANCOVA analyses were performed using SPSS Statistics software (version 23, IBM Corp., Armonk, NY, USA). Statistical significance was set at *P* < 0.05. The data are presented as means ± standard deviation.

## 3 RESULTS

### 3.1 Training completion rate in the training group

The completion rate in the training group was 100%.

### 3.2 Body composition, ${\dot{V}}_{O_{2}\max}$ and blood parameters

The percentage body fat was significantly decreased in the training group after the intervention period (*P* < 0.001). However, other body composition parameters did not change before or after the intervention period in either group.

Before the intervention period, ${\dot{V}}_{O_{2}\max}$ was similar between groups (*P* = 0.608). However, after the intervention period, the training group exhibited a significant increase in ${\dot{V}}_{O_{2}\max}$ (*P* < 0.001), which was also significantly higher compared with that of the control group (*P* = 0.018).

No significant changes were observed in plasma NOx and plasma ET‐1 concentrations across the intervention period, and no differences between groups were detected at any time point (Table 1).

### 3.3 Haemodynamic responses to sitting

No significant changes in popliteal artery diameter were observed before or after intervention in either group. No significant differences between the groups were observed at any time point. Popliteal artery blood flow, shear rate and vascular conductance were significantly decreased during the sitting period compared with the pre‐sitting level in both groups (*P* < 0.001). Moreover, no statistically significant differences were observed before or after the intervention period. Mean arterial pressure was significantly increased in response to sitting before and after the intervention period in both groups (*P* < 0.001; Table 2).

**TABLE 2:** Popliteal artery haemodynamic parameters measured before, during, and after sitting.

				Duration of sitting				Three‐way ANOVA
			Pre‐sit	1 h	2 h	3 h	Post‐sit	
Basal diameter (cm)	Control	0 weeks	0.44 ± 0.06	0.44 ± 0.03	0.43 ± 0.03	0.42 ± 0.03	0.44 ± 0.06	Period × Group: *P* = 0.17 Sitting × Group: *P* = 0.72 Period × Sitting: *P* = 0.20 Period × Sitting × Group: *P* = 0.39
		8 weeks	0.45 ± 0.05	0.42 ± 0.03	0.44 ± 0.03	0.43 ± 0.03	0.45 ± 0.05	
	Training	0 weeks	0.45 ± 0.05	0.42 ± 0.04	0.42 ± 0.05	0.42 ± 0.05	0.45 ± 0.05	
		8 weeks	0.44 ± 0.05	0.43 ± 0.03	0.44 ± 0.03	0.42 ± 0.03	0.42 ± 0.06	
Shear rate (s^−1^)	Control	0 weeks	99.7 ± 64.6	28.6 ± 14.2^*^	27.7 ± 14.5^*^	28.1 ± 22.6^*^	85.8 ± 33.6	Period × Group: *P* = 0.26 Sitting × Group: *P* = 0.95 Period × Sitting: *P* = 0.42 Period × Sitting × Group: *P* = 0.42
		8 weeks	107.5 ± 52.2	23.3 ± 11.8^*^	21.7 ± 13.0^*^	21.5 ± 8.7^*^	73.3 ± 18.8	
	Training	0 weeks	102.5 ± 39.3	31.6 ± 14.9^*^	26.2 ± 22.0^*^	35.1 ± 19.8^*^	71.7 ± 21.0	
		8 weeks	108.0 ± 27.9	38.6 ± 13.2^*^	25.3 ± 10.7^*^	21.5 ± 10.2^*^	82.2 ± 40.9	
Blood flow (mL min^−1^)	Control	0 weeks	43.0 ± 22.1	19.5 ± 9.4	18.7 ± 9.2	16.4 ± 8.9^*^	39.5 ± 13.3	Period × Group: *P* = 0.56 Sitting × Group: *P* = 0.64 Period × Sitting: *P* = 0.01 Period × Sitting × Group: *P* = 0.22
		8 weeks	58.0 ± 30.7	16.6 ± 9.3^*^	17.2 ± 10.5^*^	14.3 ± 7.1^*^	34.5 ± 13.5	
	Training	0 weeks	50.4 ± 24.5	22.6 ± 14.5^*^	17.1 ± 7.6^*^	24.1 ± 13.8^*^	33.6 ± 12.0	
		8 weeks	52.8 ± 18.1	24.9 ± 8.6^*^	17.2 ± 7.2^*^	13.5 ± 6.2^*^	31.3 ± 15.8	
Vascular conductance (mL min^−1^ mmHg^−1^)	Control	0 weeks	0.51 ± 0.26	0.21 ± 0.09^*^	0.21 ± 0.11^*^	0.18 ± 0.09^*^		Period × Group: *P* = 0.37 Sitting × Group: *P* = 0.80 Period × Sitting: *P* = 0.01 Period × Sitting × Group: *P* = 0.13
		8 weeks	0.69 ± 0.38	0.18 ± 0.10^*^	0.19 ± 0.11^*^	0.16 ± 0.07^*^		
	Training	0 weeks	0.59 ± 0.30	0.25 ± 0.16^*^	0.18 ± 0.08^*^	0.25 ± 0.14^*^		
		8 weeks	0.61 ± 0.21	0.27 ± 0.09^*^	0.19 ± 0.07^*^	0.14 ± 0.07^*^		
Mean arterial pressure (mmHg)	Control	0 weeks	86.7 ± 7.2	91.9 ± 11.8	90.1 ± 10.1	92.3 ± 12.0^*^		Period × Group: *P* = 0.17 Sitting × Group: *P* = 0.39 Period × Sitting: *P* = 0.98 Period × Sitting × Group: *P* = 0.61
		8 weeks	85.2 ± 8.7	89.4 ± 9.3	89.5 ± 10.0	88.3 ± 12.7		
	Training	0 weeks	86.8 ± 6.6	91.9 ± 8.3	94.4 ± 7.5^*^	94.9 ± 5.7^*^		
		8 weeks	86.9 ± 6.7	92.4 ± 6.9^*^	92.6 ± 8.6	95.7 ± 11.4		

Data are means ± SD. *P < 0.05 vs. Pre‐sit.

**FIGURE 1:**
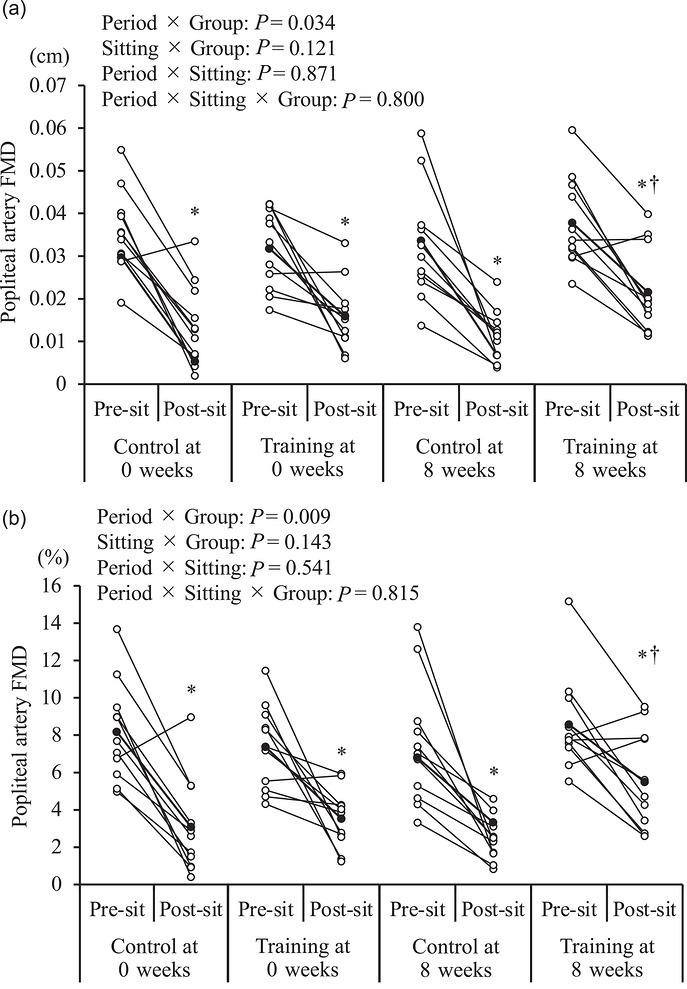
Group means and individual values for popliteal artery absolute (a) and relative (b) FMD before (pre‐sitting) and after (post‐sitting) the sitting period, measured before and after the intervention. (a) The absolute change in arterial diameter from baseline to peak dilation, (b) relative FMD (%), calculated as the percentage change from baseline diameter. Filled circles indicate group means and open circles indicate individual data. *P < 0.05 vs. pre‐sit. †*P* < 0.05 vs. post‐sit in the control at week 8.

Before the intervention period, popliteal artery absolute (cm) and relative (%) FMD were significantly decreased in post‐sitting for both groups (*P* < 0.001). Following the intervention, the pre‐sitting popliteal artery FMD did not change significantly in either group. As observed before the intervention period, popliteal artery FMD again decreased significantly in response to sitting in both groups following the intervention period. However, post‐sitting popliteal artery FMD was significantly higher in the training group compared with that in the control group (*P* = 0.003; Figure 1). This difference was supported by a significant session × group effect (*P* = 0.034, partial η^2^ = 0.206).

**FIGURE 2:**
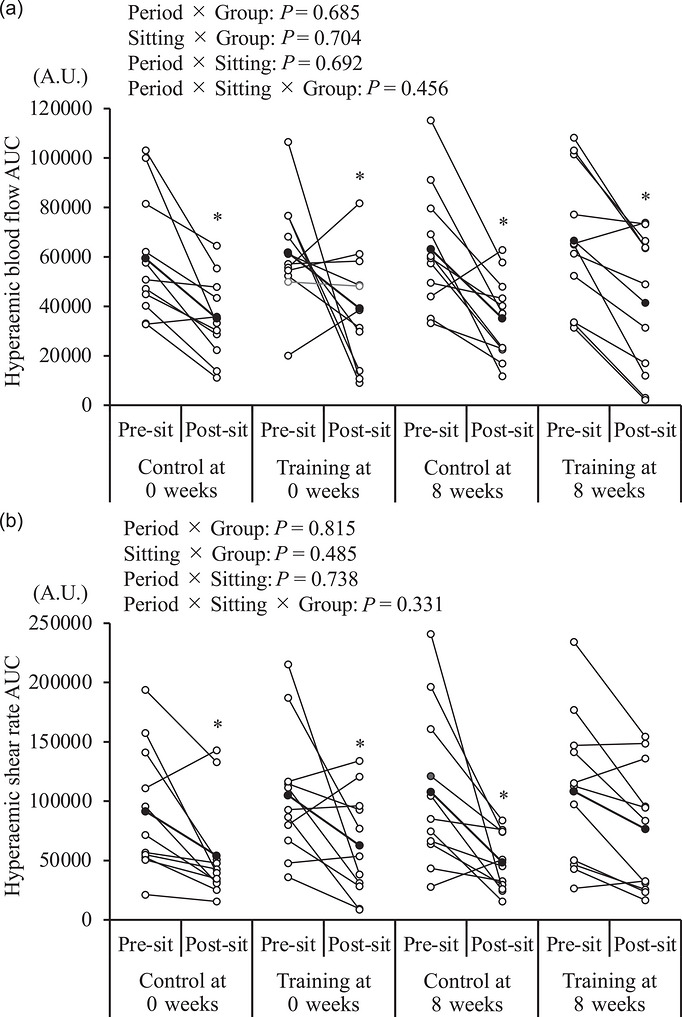
Group means and individual values for hyperaemic blood flow (a) and shear rate (b) area under the curve (AUC) before (pre‐sitting) and after (post‐sitting) the sitting period, measured before and after the intervention. Filled circles indicate group means and open circles indicate individual data. *P < 0.05 vs. pre‐sit.

Hyperaemic blood‐flow AUC was significantly decreased from pre‐sitting to post‐sitting in both groups before and after the intervention period (*P* < 0.001, η^2^ = 0.556). Although the hyperaemic shear rate AUC was significantly decreased from pre‐sitting to post‐sitting in both groups before the intervention period (*P* < 0.001, η^2^ = 0.582), the training group did not exhibit a significant reduction in shear rate AUC following sitting post‐intervention (*P* = 0.056; Figure 2).

To control for the potential influence of pre‐sitting popliteal artery FMD in the intervention period, an ANCOVA was performed, using pre‐sitting FMD at week 8 as the covariate. The results indicated that the between‐group difference in post‐sitting popliteal artery FMD was significant (*P* = 0.006, η^2^ = 0.337), suggesting that the effect could not be explained by baseline FMD levels alone. Similarly, ANCOVA was performed using post‐sitting blood‐flow AUC, shear rate AUC and basal diameter after the intervention period as covariates. The group differences in post‐sitting FMD were statistically significant (blood‐flow AUC; *P* = 0.005, η^2^ = 0.352, shear rate AUC; *P* = 0.012, η^2^ = 0.287, basal diameter; *P* = 0.006, η^2^ = 0.332; Table 3).

**TABLE 3:** ANCOVA of post‐sitting FMD adjusted for pre‐sit FMD, post‐sit blood flow AUC, shear rate AUC and basal diameter after the intervention period.

	df	*F*	*P*	Partial η^2^
Covariate: pre‐sit FMD at 8 weeks				
Group	1	9.646	0.006	0.337
Pre‐sitting FMD	1	1.284	0.271	0.063
Error	18			
Covariate: post‐sit blood flow AUC at 8 weeks				
Group	1	10.303	0.005	0.352
Post‐sit blood flow AUC	1	0.849	0.368	0.043
Error	18			
Covariate: post‐sit shear rate AUC at 8 weeks				
Group	1	7.645	0.012	0.287
Post‐sit SRAUC	1	2.927	0.103	0.134
Error	18			
Covariate: post‐sit basal diameter at 8 weeks				
Group	1	9.423	0.006	0.332
Post‐sit basal diameter	1	0.457	0.507	0.023
Error	18			

Analysis of covariance (ANCOVA) was conducted to compare post‐sit FMD between groups, adjusting for pre‐sit FMD, post‐sit blood flow AUC, shear rate AUC and basal diameter after the intervention period.

## 4 DISCUSSION

The major findings of the present study were as follows. (1) Three hours of sitting significantly decreased popliteal artery FMD and hyperaemic blood flow AUC in both groups, before and after the intervention period. (2) After the intervention, post‐sitting FMD in the training group was significantly higher compared with that in the control group. (3) This between‐group difference in post‐sitting FMD remained statistically significant after adjusting for pre‐sitting FMD. It also persisted after adjustment for post‐sitting hyperaemic blood‐flow and shear rate AUC at week 8. The results indicate that 8 weeks of HIIT does not prevent sitting‐induced endothelial dysfunction nor preserve microvascular hyperaemia.

In the present study, despite a significant improvement in aerobic capacity by HIIT, 3 h of sitting still resulted in marked endothelial dysfunction. This underscores the substantial vascular stress imposed by prolonged sitting, which may not be fully counteracted by short‐term HIIT. The discrepancy observed in our previous study of competitive cyclists (Morishima et al., 2020b) likely reflects differences in aerobic fitness. In the present study, ${\dot{V}}_{O_{2}\max}$ was 49.3 ± 4.9 mL kg^−1^ min^−1^ after training, whereas the cyclists exhibited 60.8 ± 3.6 mL kg^−1^ min^−1^. Consistently, a study spanning a wide fitness range reported sitting‐induced FMD decreases at approximately 50 mL kg^−1^ min^−1^ but not at 60 mL kg^−1^ min^−1^ (Liu et al., 2021), and similar declines across fitness levels have been reported more recently (Daniele et al., 2026). It is therefore possible that the ∼6 mL kg^−1^ min^−1^ improvement in aerobic capacity observed in the present study was insufficient to meaningfully alter vascular responses to prolonged sitting. Larger or longer‐term improvements in aerobic fitness may be required. Even in generally active adults, minimizing uninterrupted sitting and incorporating walking breaks may be necessary to preserve vascular health.

Regarding the microvascular responses, our results do not support preservation after training. At week 8, hyperaemic blood‐flow AUC decreased after sitting in the training group, and the shear rate AUC also decreased with sitting before and after intervention. Our previous study of competitive cyclists (Morishima et al., 2020b) similarly showed a reduction in hyperaemic blood‐flow AUC after sitting (*P* = 0.058), together with a significant reduction in shear rate AUC. This indicates that uninterrupted sitting depresses the microvascular hyperaemic stimulus over a range of aerobic fitness levels. Similar sitting‐induced reductions in microvascular haemodynamics have also been reported in both high‐ and low‐fit individuals, including declines in limb blood flow, shear rate and tissue oxygenation during prolonged sitting (Daniele et al., 2026). Although shear rate AUC and FMD are strongly correlated in young adults (Pyke & Tschakovsky, 2005, 2007; Pyke et al., 2004), the between‐group difference in post‐sitting FMD at week 8 persisted after adjusting for post‐sitting shear rate AUC (ANCOVA, *P* = 0.012). Together with the observed decrease in microvascular indices, these findings indicate that differences in microvascular hyperaemia do not account for the higher post‐sitting FMD in the training group at week 8. Mechanistically, prolonged sitting produces a low‐shear, high‐resistance milieu in the lower limb, which is driven by muscle‐pump inactivity (Morishima et al., 2016; Tikkanen et al., 2013), arterial angulation at the knee (Walsh et al., 2017), and sympathetic vasoconstrictor effects (Notarius et al., 2023). Under these conditions, popliteal artery blood flow and shear rate fall, which likely suppresses the downstream hyperaemic response (Morishima et al., 2016; Restaino et al., 2016). Exercise training can induce microvascular adaptations, such as increased capillary density and greater endothelial eNOS content (Cocks et al., 2013, 2016); training‐induced upregulation of endothelial NO signalling has also been described (Green et al., 2004, 2017). These adaptations appear dose‐ and compartment‐specific, and the 8‐week HIIT used in the present study was insufficient to normalize sitting‐induced deficits in reactive hyperaemia. Consistent with this interpretation, plasma NOx remained unchanged after training, which indicated that NO bioavailability did not improve and was insufficient to counteract the low‐shear conditions resulting from prolonged sitting. Plasma ET‐1 was also unchanged, and plasma levels of these mediators may not reflect local endothelial signalling. Taken together, these observations indicate that microvascular function was not maintained after HIIT and that the modest mitigation of the sitting‐induced FMD decrease is consistent with conduit‐artery adaptations compared with the preservation of downstream microvascular hyperaemia.

There are several limitations in the present study. First, the modest sample size may limit power to detect small effects or interaction terms. Therefore, null findings should be interpreted with caution. Second, menstrual cycle phase was not recorded for female participants and therefore could not be accounted for in the analyses, which should be considered when interpreting the findings. Future studies should further examine potential sex‐specific responses to prolonged sitting and HIIT, with careful control of menstrual cycle phase and hormonal status.

In conclusion, 8 weeks of HIIT did not prevent the sitting‐induced decrease in popliteal artery FMD. Microvascular hyperaemia also decreased after sitting at week 8, as indicated by reductions in blood‐flow AUC, and these changes were similar in both groups. Nevertheless, post‐sitting FMD at week 8 was higher in the training group than in the control group, even after adjustment for pre‐sitting values, indicating conduit‐artery adaptations without preservation of microvascular function. These results indicate that the vascular burden imposed by uninterrupted sitting is substantial and cannot be fully countered by exercise training alone, underscoring the need to combine regular vigorous exercise with strategies that interrupt or reduce prolonged sitting to more effectively preserve lower‐limb vascular health.

## AUTHOR CONTRIBUTIONS

Nobukazu Kasai and Takuma Morishima conceptualized and designed the study. Nobukazu Kasai, Hayato Ihara, and Takuma Morishima performed experiments. Hayato Ihara and Takuma Morishima analysed data. Nobukazu Kasai, Motoyuki Iemitsu, and Takuma Morishima interpreted the results of experiments. Takuma Morishima prepared figures and tables. Takuma Morishima drafted the manuscript. Nobukazu Kasai, Hayato Ihara, Motoyuki Iemitsu, and Takuma Morishima edited and revised the manuscript. All authors have read and approved the final version of this manuscript and agree to be accountable for all aspects of the work in ensuring that questions related to the accuracy or integrity of any part of the work are appropriately investigated and resolved. All persons designated as authors qualify for authorship, and all those who qualify for authorship are listed.

## CONFLICT OF INTEREST

No conflicts of interest, financial or otherwise, are declared by the authors.

## Data Availability

These data have not been made publicly available. However, the corresponding author can provide further information on the data upon reasonable request.
